# A within-person investigation of recovery identity following substance use disorder: examining the impact of recovery-focused social contexts

**DOI:** 10.3389/fpubh.2025.1534432

**Published:** 2025-02-12

**Authors:** Joseph H. Lancaster, Hannah B. Apsley, Timothy R. Brick, H. Harrington Cleveland

**Affiliations:** ^1^Department of Human Development and Family Studies, The Pennsylvania State University, University Park, PA, United States; ^2^Institute for Computational and Data Sciences, The Pennsylvania State University, University Park, PA, United States

**Keywords:** recovery identity, dynamic, meaningfulness, substance use disorder, recovery meeting, social context, within-person, random effects Tobit model

## Abstract

**Background:**

The social identity model of recovery (SIMOR) posits that adopting a recovery identity is vital for achieving favorable recovery outcomes. Until now, no studies have investigated recovery identity as a dynamic construct, although recent findings suggest it fluctuates from one day to the next. The present study examines the within-person association between recovery identity and sense of meaningfulness—an aspect of holistic recovery wellbeing. Because recovery-focused social contexts exist to support individuals’ recovery wellbeing, we assessed the moderating impact of two such contexts (recovery community centers [RCCs] and recovery meetings) as same-day moderators.

**Methods and materials:**

91 RCC visitors across Pennsylvania completed daily diary surveys for 10 evenings. Daily measures of recovery identity, meaningfulness, recovery meeting and RCC attendance were analyzed in a multilevel Tobit model (to address right-censoring in the outcome data).

**Results:**

Results indicated both day-level recovery identity (*b* = 0.79, *SE* = 0.04, *p* < 0.001) and person-level recovery identity (*b* = 0.94, *SE* = 0.11, *p* < 0.001) were positively associated with daily meaningfulness. Although the day-level interaction with RCC attendance was not significant (*b* = −0.11, *SE* = 0.14, *p* = *n.s.*), the interaction with recovery meeting attendance was (*b* = −0.27, *SE* = 0.13, *p* = 0.039), suggesting that meeting attendance buffered the effect of recovery identity on meaningfulness. A simple slopes analysis indicated that the relationship of recovery identity with meaningfulness was still statistically significant and positive in both cases (attended: *b* = 0.56, *SE* = 0.08, *p* < 0.001; not attended: *b* = 0.87, *SE* = 0.06, *p* < 0.001).

**Conclusion:**

These results suggest that people reporting stronger recovery identity also reported greater day-to-day meaningfulness. Further, on any given day for an individual, meaningfulness was higher on days recovery identity was stronger than usual for that individual, and lower on days when recovery identity was weaker. Meeting attendance reduced this effect, suggesting that meeting attendance may be especially helpful to recovery on days when recovery identity is low.

## 1 Introduction

Achieving recovery from substance use disorders (SUDs) is a challenging process. In 2022, an estimated 22.2 million adults in the United States self-identified as in recovery or recovered from problematic substance use (1), and the majority of adults with past substance use problems report that it took multiple serious recovery attempts before their problem was resolved (2). Non-professional recovery supports such as recovery community centers (RCCs) and mutual help groups (e.g., Alcoholics Anonymous, Self-Management and Recovery Training) are designed to help individuals negotiate the challenges to recovery and to help the millions of people pursuing and maintaining recovery form supportive social connections with like-minded peers. Participation in these support services has been associated with positive recovery outcomes, such as longer abstinent time (3, 4) and greater psychological wellbeing (3). The social identity model of recovery [SIMOR; (5)] posits that the success of mutual help groups and RCCs may be due to individuals forming social identities based on the recovery social group (or adopting a “recovery identity”). Personal recovery identity is critical in achieving successful recovery outcomes (5).

The present study examined the within-person association between recovery identity and meaningfulness among individuals in recovery, also assessing the moderating role of individuals’ same-day visits to recovery-focused social contexts (e.g., RCCs and mutual help group meetings) on this effect. This study used naturalistic, intensive longitudinal methods to assess daily recovery experiences in the context in which they occur. In response to a call for addiction research to move away from consumption-related outcomes (i.e., relapse) and consider recovery through a more holistic lens that embraces general wellbeing (6, 7), this study identified daily sense of meaningfulness as an outcome of interest.

SIMOR is based in social identity theory and self-categorization theory (8, 9) and subscribes to the notion that individuals’ social identities are predicated on the norms, beliefs, and attitudes of the social group to which they belong. In the context of SUD recovery, this means that belonging to a social group that is in active addiction would promote adopting a “substance-user identity,” while membership in a group of individuals who are in recovery (e.g., 12-step fellowship) would promote adopting a “recovery identity” (10). Transitioning from active addiction to a recovery identity improves multiple indices of recovery such as decreased instances of lapses and substance appetitive behavior, and greater quality of life and commitment to maintaining recovery (11–14).

To date, studies on the effects of recovery identity on recovery outcomes have primarily employed cross-sectional designs, which can only assess between-person relationships. However, social identities are inherently dynamic and correspond with changes in group membership (15); prior work suggests that this is also true of recovery identity. In a daily diary study of recovery experiences, recovery identity varied substantially from day to day, and within-person variance accounted for 43% of the variance in the recovery identity variable (16). Intensive longitudinal methods, such as daily diary studies, are able to assess both between- and within-person variation, and also confer improved ecological validity over cross-sectional designs (17). Because recovery identity varies within person, intensive longitudinal designs are needed to assess the possible within-person effects of recovery identity on daily recovery processes.

The outcome examined in the present study is meaningfulness. This concept overlaps substantially with—but is not a direct daily corollary of—“meaning in life,” which has been defined as “the extent to which people comprehend, make sense of, or see significance in their lives, accompanied by the degree to which they perceive themselves to have a purpose, mission, or over-arching aim of life” [(18), p. 682]. Meaningfulness, as conceptualized and assessed herein, is less broad both conceptually and temporally. It is operationalized around how purposeful and satisfying respondents perceived their day to be. It does not assess how people make sense of or see significance in their lives [see (18)]. Nonetheless, the concept of “meaning in life” is very relevant to this inquiry. Meaning in life has been viewed as a component of or major contributor to personal wellbeing. In SUD recovery research there has been a push to conceptualize recovery more holistically, such as by including aspects of personal wellbeing (6, 7).

Moreover, like recovery identity, meaningfulness as conceptualized herein is suitable for dynamic examination, as it may fluctuate from day to day. Two prior intensive longitudinal studies, one using the data examined here, found substantial portions of the variance in meaningfulness scores to be attributable to within-person changes [48% in (19) and 61% in (16)], rather than between-persons. Because recovery identity stems from membership in a social group and sense of belonging (to a social group) enhances meaningfulness (20), we anticipated finding a positive within-person association between recovery identity and meaningfulness.

Recovery identity generally corresponds with the feeling that someone belongs to the recovery community or feels like they are “in recovery.” Some data indicate, however, that the intensity of that identity may change from day to day and suggests recovery identity is stronger on days there is direct social contact with others in recovery, such as at an RCC (16). Given the role social contact with recovery members may play in the intensity of daily recovery identity, we wanted to examine the potential impact of participation at peer-based recovery support services on the within-person association between recovery identity and meaningfulness in an exploratory analysis. We specifically assessed two peer-based recovery support settings, RCCs and recovery meetings, and examined daily attendance at each as moderators.

Mutual-help recovery meetings are very popular sources of recovery support, and are utilized by an estimated 45.1% of U.S. adults in recovery (or, roughly 10 million people) (21). One of the mechanisms of behavior change for Alcoholics Anonymous specifically (the most popular of the mutual help meetings) is facilitating a social network change, such that individuals in recovery can meet and befriend other individuals in recovery, and reduce social ties with pro-drinking peers (22, 23). RCCs are recovery hubs run by recovery professionals and peers, often on a voluntary basis (24). Although they provide formal services, such as employment assistance and recovery coaching (25), RCCs also provide informal support akin to a social club for recovery members to socialize and connect with a recovery community.

Data were drawn from a daily diary study that sampled regular visitors of local RCCs. This study assessed two specific research aims:

**RA1:** To investigate the same-day association between end-of-day reports of recovery identity and meaningfulness.

**H1:** We hypothesized that, on days recovery identity was stronger than one’s average, sense of meaningfulness will also be greater.

**RA2:** To assess RCC and recovery meeting attendance as day-level moderators of the above association to examine whether direct social contact with recovery-supportive peers strengthens or weakens the relationship between daily recovery identity and meaningfulness. As this part of the analysis is exploratory, we do not offer directional hypotheses for how each moderator might impact the day-level association between recovery identity and meaningfulness.

## 2 Materials and methods

### 2.1 Recruitment and procedures

Participants (*N* = 94) were visitors of six RCCs in the state of Pennsylvania. Recruitment occurred through partnership with RCC leaders and staff, who distributed fliers and emails about the study. Eligibility criteria included (1) being at least 18 years of age, (2) speaking English, and (3) being otherwise able to complete the study protocol. Participants were excluded from analyses if they did not report being in recovery from a SUD.

Several research staff members met in person with interested individuals on a preplanned recruitment and on-boarding day at each RCC. After reviewing study procedures as a group, potential participants were encouraged to ask questions to study team members, either asking the main presenter or calling over other research team members to ask questions individually. All participants provided individual consent prior to their involvement. While on-site, participants completed a pen and paper baseline survey and then received instruction on how to install the Wear-IT data collection app (26) onto their smartphones and complete the daily diary protocol. Daily diary surveys were delivered for 10 consecutive evenings at 8:30 pm, with reminder notifications sent every 30 min for 2 hr. Daily diary surveys took approximately 10 min to complete. Participants were compensated in the form of Amazon gift cards, with $10 paid for completing the baseline survey and $6 paid for completing each daily diary survey. All study procedures were approved by a university internal review board.

### 2.2 Measures

#### 2.2.1 Daily meaningfulness

Meaningfulness was assessed each evening with five items using a continuous touchpoint scale (0 = Strongly disagree, 100 = Strongly agree). The items were “My day has been… `Meaningful’, `Gratifying’, `Fulfilling’, `Purposeful’, and `Satisfying’.” The variable was calculated by averaging the five daily items in the scale, such that higher scores indicated greater meaningfulness. This scale has been previously used in a daily diary study sampling individuals in SUD treatment (19). The Cronbach’s alpha for these five items was 0.96, indicating high reliability across all days and participants. The variance components from a random effects ANOVA on the items were used to calculate another reliability coefficient, R_c_, (see (27)), interpreted on a scale similar to Cronbach’s alpha. For this scale, R_c_ was 0.93, suggesting that this measure reliably reflects within-person change across study days.

#### 2.2.2 Daily recovery identity

Recovery identity was assessed at the end of each day using a composite of nine items, each with a continuous touchpoint scale with anchors at each end (0 = Strongly disagree, 100 = Strongly agree). The nine items were: “Thinking about today, I feel like… `I was committed to my recovery’, `I worked hard on my recovery’, `I kept my recovery central to my day’, `I felt like a “person in recovery”’, `I felt connected to other people in recovery’, `I was grateful to be in recovery’, `I thought of myself as being part of the recovery community, even when I was not with other people in recovery’, `My being in recovery guided my decisions’, `I missed my old drug use/ drinking social group’” (reverse coded). The variable was calculated by averaging the nine daily items in the scale, such that higher scores indicated stronger recovery identity (*α* = 0.91; R_c_ = 0.86), and then decomposed into day- and person-level variables in preparation for multilevel models. The person-level variable was calculated by taking the mean of each participant’s 10 daily scale scores, reflecting each participant’s average degree of recovery identity across the study period. This allowed us to control for the between-person effect and isolate the within-person effect of recovery identity. The day-level variable was person-mean centered by subtracting participants’ mean scores from each of their observed scores (28, 29).

#### 2.2.3 Daily recovery community center attendance

RCC attendance was assessed each night with one item, “Where did you spend time today?” with “Recovery Community Center” listed as one of several response options (instructions specified “check off all that apply”). The “Recovery Community Center” option was then coded into a binary variable (0 = did not attend RCC, 1 = attended RCC). RCC attendance was then decomposed into day- and person-level variables, with day-level RCC attendance person-mean centered (28, 30). The resulting day-level variable represents a tendency-weighted effect of daily visitation, so that a person who visits the RCC every day contributes to the person-level effect but not to the day-to-day effect (because they show no day-to-day differences). This effect then represents the difference between going to the RCC and not going to the RCC, controlling for the person’s overall tendency to go to the RCC, and can be interpreted as a generalized effect of “uncharacteristically” going to or missing the RCC. To compute the person-level variable, each participant’s count of daily RCC visits was averaged across the 10 study days, reflecting the proportion of study days that each participant visited the RCC.

#### 2.2.4 Daily recovery meeting attendance

Recovery meeting attendance was operationalized via two items that asked about activities in and outside of participants’ RCCs. The items were, “Which of the following activities/services did you take part in at [or ‘outside’] your RCC today? (Please check off all that apply),” respectively. Both items contained the response option “Attended a recovery support group meeting (e.g., 12-step or any other group meeting),” from which a binary variable was then created (0 = did not attend a recovery meeting, 1 = attended a recovery meeting). Recovery meeting attendance was then decomposed into variables at the day and person level. Day-level variables were person-mean centered (28, 30) with the same interpretation as for RCC attendance. The person-level variable was created by averaging each participant’s count of daily recovery meetings attended (up to one per day) across the 10 study days, reflecting the proportion of study days that each participant went to a recovery meeting. When participants responded affirmatively to having attended a recovery meeting, they were then presented with a follow-up item regarding which type of meeting they attended (e.g., AA, NA, etc.). Most of the recovery meetings attended during the study were 12-step groups (84%).

#### 2.2.5 Covariates

To account for any potential between-person demographic differences in meaningfulness, we examined age, biological sex, and race as covariates. Age was a continuous variable calculated from self-reported birth dates; sex (0 = male, 1 = female) and race (0 = White, 1 = Asian, 2 = Black or African American, 3 = Native Hawaiian or other Pacific Islander, 4 = American Indian or Alaska Native, 5 = multiracial, 6 = other) were categorical variables. In addition to biological sex, we also assessed gender, with the options of male, female, transgender, and non-binary. As no individuals endorsed transgender or non-binary, responses to this item were identical to the biological sex item. A day of study variable (1–10) was also created to reflect the passage of time since the study’s onset. We considered time as a potential confound because being prompted to reflect on daily events (as is done with the end-of-day surveys) may increase feelings of meaningfulness over time (31).

### 2.3 Data analysis

A multilevel modeling framework was used to examine recovery identity and its interactions with RCC and recovery meeting attendance as same-day predictors of meaningfulness, adjusting for person-level main effects and other confounds (40).

#### 2.3.1 Data inspection

Because the data came from a repeated measures design (i.e., daily diary) and may have within-participant dependence, intraclass correlation coefficients (ICCs) were calculated for day-level variables. The ICC for meaningfulness, recovery identity, and RCC and recovery meeting attendance were 0.39, 0.57, 0.09, and 0.27, respectively. The ICC indicates that, of the total variance in recovery identity, for instance, 57% is attributable to between-person variation, leaving 43% of the variance to be within individuals (across days). Coefficients in this range warrant the use of multilevel modeling (27).

In checking model assumptions (i.e., normality), a ceiling effect was detected in the meaningfulness data (125 observations at the maximum value—100). This results in *right-censored* data, where values that would be over 100 are all reported as 100. In other words, this bunching of responses at the maximum value suggests that there is variability in the phenomenon that was not being fully accessed; and thus, was “censored.” To address the right-censored (and nested) outcome, random effects Tobit (censored regression) models were fit. A Tobit model is a two-part joint regression model which provides parameter estimates that can be interpreted like linear regression by assuming that the true scores of the censored data make the outcome’s full distribution Gaussian (i.e., a latent normal distribution (32)). Put differently, random effects Tobit models specify a mixed-effects regression in which the dependent variable is latent (partially unobserved), and all independent variables are manifest or observable [(33); for more information and an example, see Ch. 9 in (34)]. Random effects Tobit models were fit with R Statistical Software [v4.2.2; (35)] using the censReg package [0.5-39; (36)] and the plm package (37) to add random effects (for details on how this was performed, see (38)). Model parameters were estimated using the BHHH method (39), with statistical significance evaluated at *α* = 0.05.

In the daily diary data (799 days nested within 94 individuals), there was minimal missing data (85% daily diary compliance). Meaningfulness data was available on 788 days (98.6% of non-missing days), recovery identity on 786 days (98.4% of non-missing days), RCC attendance on 793 days (99.3% of non-missing days), and recovery meeting attendance on all 799 reported days (100%). Three participants were excluded from analyses because they did not have complete data (3.5% missing days), leaving a final set of 771 days across 91 individuals.

#### 2.3.2 Model building

Potential confounds were considered through the model building process. Models were compared and selected based on chi-square goodness of fit tests for nested models and based on AIC/BIC fit statistics for non-nested models. First, the covariates listed above (age, sex, race, and day of study) were examined independently as predictors of meaningfulness. Age, race, and day of study (but not sex) were statistically significant; sex was dropped from subsequent models. Next, we examined the main and interaction effects of recovery identity, RCC attendance, and recovery meeting attendance, including the interaction between day- and person-level recovery identity (although not retained). At this stage, the model included age, race, day of study, day- and person-level recovery identity, day- and person-level RCC attendance, day- and person-level recovery meeting attendance, and the day-level interactions for recovery identity with RCC attendance and recovery identity with recovery meeting attendance—all as predictors of daily meaningfulness. During the building process, all models included random intercepts but could not accommodate random slopes due to software limitations. All slope estimates are therefore fixed to be the same for all individuals across the sample. All models converged successfully. *Post hoc* analyses involved examining the simple slopes of significant interactions.

For the final random effects Tobit model, the measurement equation is as follows:


$$y_{pd}=\{\begin{array}{c} y_{pd}^{*}\;\mathrm{if}\;y_{pd}^{*}<y_{U},\\ y_{U}\;\;\mathrm{if}\;y_{pd}^{*}\geq y_{U}\text{.} \end{array}$$

where $y_{pd}$ is an observable variable; $y_{U}$ represents the point at which data are right-censored (100); and $y_{pd}^{*}$ represents a normally distributed latent (unobservable) variable that is identical to the observable variable for uncensored scores (<100).

The structural equation for that model is:


ypd∗=γ00+γ01MeanRecoveryIdentityp+γ10RecoveryIdentitypd+γ02MeanRCCp+γ20RCCpd+γ03MeanRecoveryMeetingp+γ30RecoveryMeetingpd+γ04Agep+γ05Racep+γ40Daypd+γ50RecoveryIdentitypd×RCCpd+γ60RecoveryIdentitypd×RecoveryMeetingpd+u0p+εpd

In the structural equation above, *_p_* denotes persons and *_d_* denotes days. At the day level, $y_{pd}^{*}$ is the observed (<100) or unobserved meaningfulness score for person *p* on day *d*. The intercept was allowed to vary by individual and could therefore be interpreted as the expected level of meaningfulness for a typical person white male who did not go to the RCC or a meeting and reported the lowest recovery identity, measured on the day before the study began. This random intercept is denoted by parameter γ_00_ representing the fixed component (that is, the mean intercept) and parameter u_0p_ representing the random component (deviation around the mean for that individual). Parameters γ_10_, γ_20_, γ_30_, and γ_40_ represent (fixed) day-level slopes, indicating within-person differences in meaningfulness associated with day-to-day differences in recovery identity, RCC attendance, recovery meeting attendance, and the day of the study, respectively; γ_50_ and γ_60_ indicate within-person interactions for recovery identity with RCC attendance and with recovery meeting attendance, respectively. Parameters γ_01_, γ_02,_ γ_03_, γ_04,_ and γ_05_ represent the strength of the association between person-average recovery identity, RCC attendance, and recovery meeting attendance, as well as baseline age and sex with meaningfulness, respectively. *ε*_pd_ are day-specific residuals.

## 3 Results

Descriptive statistics and correlations for study variables are provided in Table 1. The demographic characteristics of the sample can be found in Table 2.

**Table 1:** Correlations and descriptive statistics for study variables between- and within-subjects.

	1	2	3	4	5	6	7	M	SD	Range
Person-level variable										
1. PM Recovery Identity	1							82.42	13.25	47.38–99.96
2. PM RCC Attendance (1 = visited)	0.06	1						0.31	0.21	0–1
3. PM RM Attendance (1 = visited)	0.18***	0.17***	1					0.38	0.29	0–1
Day-level variable										
4. Day of Study	0.02	−0.02	−0.02	1				4.41	2.85	1–10
5. Recovery Identity	0.78***	0.04	0.14***	0.02	1			82.47	16.92	1.78–100
6. RCC Attendance (1 = visited)	0.02	0.45***	0.07*	−0.26***	0.12***	1		0.31	0.46	0–1
7. RM Attendance (1 = visited)	0.11**	0.10**	0.60***	−0.05	0.16***	0.24***	1	0.38	0.49	0–1
8. Meaningfulness	0.51***	−0.04	0.08*	0.08	0.65***	0.07*	0.13***	78.89	21.04	0–100

PM, Person-mean; RCC, Recovery community center;. RM, Recovery meeting; M, Mean; SD, Standard deviation. *** = *p* < 0.001. ** = *p* < 0.01. * = *p* < 0.05.

**Table 2:** Demographic characteristics of the sample (*N* = 91).

	M (SD)/*N* (%)
Age (in years)	43.65 (12.34)
Sex = Female	48 (52.8%)
Race	
American Indian/Alaska Native	1 (1.1%)
Black/African American	14 (15.4%)
White	71 (78%)
Multiracial	3 (3.3%)
Other	2 (2.2%)
Hispanic Ethnicity	1 (1.1%)
Annual Household Income	
Less than $10,000	21 (23.1%)
$10,000 to $24,999	25 (27.5%)
$25,000 to $49,999	20 (22%)
$50,000 to $74,999	11 (12.1%)
$75,000 or more	14 (15.4%)
Education	
Less than high school	2 (2.2%)
Some high school	6 (6.6%)
High school diploma or GED	31 (34.1%)
Some college, no degree	23 (25.3%)
Associate degree or equivalent	11 (12.1%)
Completed trade or professional school	2 (2.2%)
Bachelor’s degree	13 (14.3%)
Master’s degree	2 (2.2%)
Greater than a Master’s degree	1 (1.1%)

### 3.1 Is recovery identity associated with higher same-day sense of meaningfulness?

Results from the random effects Tobit model indicated that recovery identity is significantly and positively related to meaningfulness at the person (between-persons: *b* = 0.94, *SE* = 0.11, *p* < 0.001) and day level (within-person: *b* = 0.79, *SE* = 0.04, *p* < 0.001), supporting our hypothesis. At the person level, these results convey that *individuals* who generally report stronger recovery identity also tend to report experiencing a greater sense of meaningfulness. At the day level, these findings mean that on *days* individuals report stronger recovery identity than their usual, they also endorse experiencing a greater sense of meaningfulness.

### 3.2 Does the strength of the same-day association between recovery identity and meaningfulness depend on whether an RCC or recovery meeting was attended that day?

**Figure 1:**
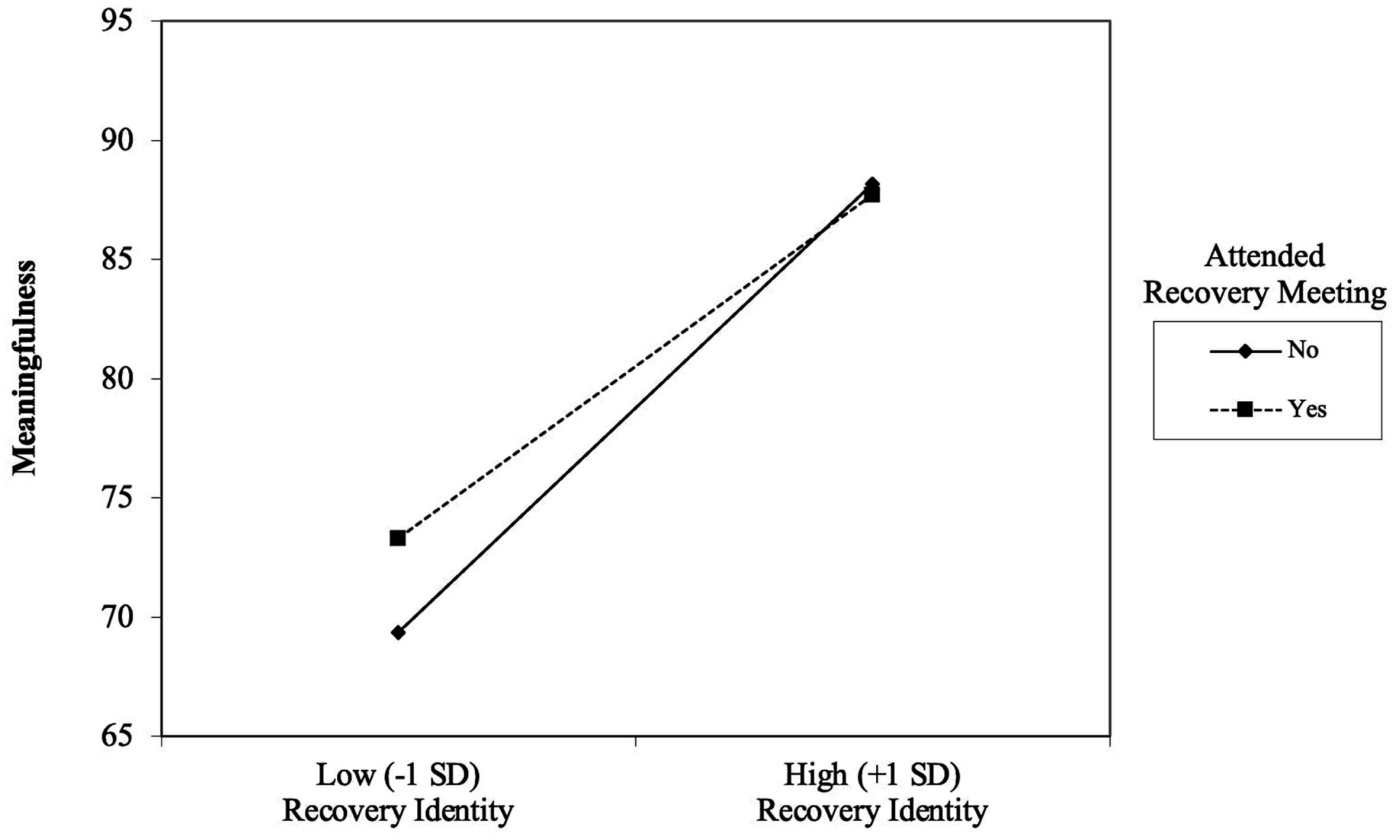
Simple slopes of daily recovery meeting attendance as a moderator of the day-level association between recovery identity and meaningfulness:

The same random effects Tobit model mentioned above also included moderators of recovery identity at the day level, those being RCC attendance and recovery meeting attendance. Results indicated that daily RCC attendance did not significantly interact with daily recovery identity in predicting daily meaningfulness (*b* = −0.11, *SE* = 0.014, *p* = 0.418). The day-level interaction between recovery identity and recovery meeting attendance, however, was statistically significant (*b* = −0.27, *SE* = 0.013, *p* = 0.041). The follow-up simple slopes analysis revealed that on days with greater meeting attendance, the association between recovery identity and meaningfulness was weaker (*b* = 0.56, *SE* = 0.08, *p* < 0.001) relative to days with lower meeting attendance (*b* = 0.87, *SE* = 0.06, *p* < 0.001). Figure 1 depicts the conditional slopes and intercepts for meeting attendance. Complete parameter estimates for the model are available in Table 3.

**Table 3:** Results of the random effects Tobit model examining the within-person association between recovery identity and meaningfulness, moderated by daily RCC and recovery meeting attendance.

Fixed Effects	Est.	S.E.	*p*
Intercept	0.76	10.51	0.942
Day of Study	0.66	0.18	<0.001
Race: American Indian/Alaska Native	7.05	190.71	0.971
Race: Black/African American	2.30	3.19	0.472
Race: Multiracial	4.21	113.17	0.97
Race: Other	−0.02	26.55	0.999
Age	0.03	0.11	0.818
PL Recovery Identity	0.94	0.11	<0.001
DL Recovery Identity (PMC)	0.79	0.04	<0.001
PL RCC Attendance	−8.62	7.72	0.264
DL RCC Attendance (PMC)	1.82	1.82	0.318
PL RM Attendance	2.98	5.23	0.568
DL RM Attendance (PMC)	2.24	1.67	0.181
DL RI × DL RCC Attendance	−0.11	0.14	0.418
DL RI × DL RM Attendance	−0.27	0.13	0.041

Random Effects	Est.	*p*
Intercept Variance	2.29	<0.001
Residual Variance	2.67	<0.001

N participants = 91; N days = 771 (646 uncensored). PL, Person-level; DL, Day-level. PMC, Person-mean centered; RCC, Recovery community center; RM, Recovery meeting; RI, Recovery identity.

## 4 Discussion

The present study used mixed-effects Tobit regression to investigate the same-day association between recovery identity and meaningfulness, considering the moderating role of day-level social contact with a recovery group through peer-based recovery supported services. The between-person finding that those with a stronger recovery identity tend to experience a greater sense of meaningfulness is consistent with SIMOR, which posits that adopting a recovery identity is critical to positive SUD outcomes (10). These findings are also consistent with other between-person research based on SIMOR (10–15). The within-person findings indicate that recovery identity varies daily and covaries positively with daily meaningfulness. These results showcase the value in studying recovery identity as a dynamic phenomenon and suggest that recovery identity may be an important intervention point to potentially impact wellbeing at the daily level.

Interestingly, we also found that meeting attendance reduced the effect of recovery identity on meaningfulness. This means that on days when recovery identity was lower than usual, meaningfulness was also lower than usual. However, if the participant attended a recovery meeting, this effect was lessened. Although the participant’s meaningfulness was still likely to be lower than usual when recovery identity was lower than usual, it would not be as much lower as on days without meeting attendance. This pattern may suggest that on difficult recovery days, marred by low recovery identity and low meaningfulness, going to a recovery meeting may be particularly beneficial, and perhaps buffer against the possible deleterious effects of not feeling connected to one’s own recovery identity.

Although other interpretations are possible, we interpret these findings to mean that when individuals struggle to feel connected with their recovery, social contact with others in recovery (in a recovery-oriented setting) can elevate their sense of meaningfulness. Of course, this line of reasoning suggests both recovery meetings and RCCs should be influential settings (and not just recovery meetings). It seems plausible that RCC attendance was not a significant moderator for statistical power reasons. RCCs were only attended on 2 of 10 days, on average, and often co-occurred with meeting attendance, making them difficult to distinguish. If it is in fact the case that RCC attendance does not show the same interactions, perhaps there are opportunities unique to recovery meetings that are not available at RCCs that are causing the disparate results. For instance, at recovery meetings, sense of meaningfulness may be boosted by listening to the stories of others who were in similar situations (known in 12-step meetings as sharing one’s “experience, strength, and hope”). Sharing testimonials is inherent to the recovery meeting experience (as it is built into the platform) and may be less common in the typical RCC experience.

### 4.1 Strengths and limitations

The primary strength of this study rests in the methodology applied. First, the use of smartphones to collect daily diary data increased the study’s ecological validity (while reducing recall bias) because the surveys were taken daily in natural environments (e.g., at home). Second, the repeated measures design allowed us to examine within-person recovery processes as they unfold over time. Given the daily variation in recovery identity, this study adds significantly to that literature, of which most findings focus on explaining between-person variation. These two methodological aspects also allow us to consider how same-day associations between variables might change based on the social contexts that were experienced.

The present findings should be interpreted in the light of some important limitations as well. First, it should be noted that all the recovering individuals in this study attend RCCs. As such, the present sample may have more severe problems with substances or be otherwise different than other individuals in recovery who do not attend RCCs. For instance, evidence of high commitment to recovery might be found in the high daily diary survey completion rate (84%). Another important consideration is that the sampling was restricted to one geographical location (Pennsylvania, USA) and, therefore, may not generalize to other contexts.

Of particular note, it is critical to clarify that the study design is observational and not experimental as this affects how the findings should be interpreted. Absent of experimental manipulation or random assignment, the present results are not causal and should be interpreted strictly as correlational associations. In other words, we cannot infer from these data that identifying as in recovery *causes* greater same-day sense of meaningfulness; it is equally possible that people are simply more likely to feel connected to their recovery when meaningfulness is higher.

## 5 Conclusion

To our knowledge, this is the first study to examine the within-person association between recovery identity and meaningfulness. We examined the impact of two recovery-focused social contexts (RCCs and recovery meetings) on the daily linkages between recovery identity and meaningfulness to explore the role of daily social contact with others in recovery. Attending daily recovery meetings but not RCCs played a significant moderating role, elevating individuals’ sense of meaningfulness on days their recovery identity was weak. Not only are these findings consistent with the idea that recovery identity is an important day-level factor in recovery wellbeing, but they also call attention to the role recovery-focused social contexts can have in coping with low recovery identity. Indeed, the present findings suggest recovery identity is an important construct in SUD intervention research and should be studied as a dynamic phenomenon. We recommend future studies examine associations with recovery identity at more intensive time intervals via multiple within-day measurement, considering that the intraindividual variation in, and effects of, recovery identity may be both momentary and context-dependent, or directly manipulate meeting attendance, perhaps with an adaptive intervention approach.

## Data Availability

The raw data supporting the conclusions of this article will be made available by the authors, without undue reservation.
